# Prediction of novel alloy phases of Al with Sc or Ta

**DOI:** 10.1038/srep09909

**Published:** 2015-05-07

**Authors:** Ante Bilić, Julian D. Gale, Mark A. Gibson, Nick Wilson, Kathie McGregor

**Affiliations:** 1 CSIRO Manufacturing, Virtual Nanoscience Lab, Parkville, VIC 3052, Australia; 2 Nanochemistry Research Institute, Department of Chemistry, Curtin University, P.O. Box U1987, Perth, WA 6845, Australia; 3 CSIRO Manufacturing, Clayton, VIC 3168, Australia; 4 CSIRO Mineral Resources, Clayton, VIC 3168, Australia

## Abstract

Using the evolutionary optimization algorithm, as implemented in the USPEX crystal predictor program, and first principles total energy calculations, the compositional phase diagrams for Al-Sc and Al-Ta alloy systems at zero temperature and pressure have been calculated. In addition to the known binary intermetallic phases, new potentially stable alloys, AlSc_3_ and AlTa_7_, have been identified in the Al-poor region of the phase diagram. The dynamic and thermal stability of their lattices has been confirmed from the calculated vibrational normal mode spectra in the harmonic approximation.

Aluminum is the most abundant metal in the Earth's solid crust. It is also the most commonly used non-ferrous metal, usually in the form of an alloy, owing largely to its low density and corrosion resistance. In recent decades intermetallics of aluminum and scandium have attracted substantial interest^1^,^2^ as a new generation of structural materials with high strength and creep resistance. The Al-Sc phase diagram^3^,^4^ exhibits three well established binary alloys, Al_3_Sc, Al_2_Sc and AlSc with cubic crystal systems and AlSc_2_ with a hexagonal lattice. The Al_3_Sc phase, with a face centered cubic (fcc) L1_2_ crystal structure^1^, has particularly desirable mechanical properties. The formation of coherent Al_3_Sc precipitates through an appropriate age-hardening heat treatment of Al-Sc alloys results in a higher strength enhancement in Al than alloying with any other element investigated to date. The homogeneously distributed nanoscale Al_3_Sc precipitates are considered to be highly effective pinning points against dislocation motion. However, Sc is sparsely distributed in the environment, naturally occurring only in trace amounts^5^. Hence, the prohibitive costs associated with Sc production necessitates the use of alternatives, such as Ti alloys, with comparable density and strength but lower cost.

Tantalum is another highly corrosion resistant element widely used as a minor component in alloys. Tantalum aluminide (Al_3_Ta), with a tetragonal D0_22_ lattice^6^,^7^,^8^,^9^, has been recognized for its potential as an advanced high-temperature structural material^10^. The crystal structure is closely related to that of the cubic L1_2_ form, which can be obtained through a substitution of a ternary component for Al^6^,^10^. In contrast with Al-Sc, however, the literature reveals rather conflicting reports regarding the Al-Ta phase equilibria data^3^,^10^,^11^,^12^. The most likely reasons behind the discrepancies are the large number of Al-Ta compounds, their structural complexity, the proximity (and overlap for non-stoichiometric concentrations) of their compositions and the difficulty in controlling the stoichiometry of samples. Thermodynamic modelling, using the CALPHAD method, seemingly only adds to the controversy^12^,^13^,^14^ surrounding the stability of the binary phases and their crystal structures. Intermetallics with compositions of Al_3_Ta, Al_2_Ta, Al_3_Ta_2_, AlTa, AlTa_2_, AlTa_3_ have been reported in those studies, in addition to the less common Al_69_Ta_39_ and Al_38_Ta_48_ stoichiometries found in more recent and accurate investigations^15^,^16^,^17^. The composition and temperature range of their stability, as well as the nature (i.e., stable vs. metastable), and atomic structure, remain largely uncertain.

The goal of the present work is to investigate the potential of evolutionary structure prediction methods for the identification of thermodynamically stable binary (and, in future, also ternary) alloy phases. To this end we set out to reproduce the composition, crystal structure and formation enthalpy of the known stable intermetallics of the Al-Sc system. After successfully generating these structures from first principles, we attempt to identify the stable phases and their crystal structures in the Al-Ta system using the same approach.

## Computational methods

Evolutionary algorithms mimic Nature's concept of the survival of the fittest to identify the optimal solution to a problem. Such algorithms have been shown to be an efficient means of searching for global minima on a complex potential energy landscape. Here we have specifically utilized such an evolutionary structure prediction algorithm to investigate the possible stable binary phases of Al with both Sc and Ta across the entire concentration range. In this work, crystal structure prediction was performed using the USPEX code^18^,^19^,^20^, based on the evolutionary algorithm developed by Oganov, Glass, Lyakhov and Zhu, which features local optimization of structures and a wide range of possible trial moves that can be tailored appropriately to the problem. To conduct the variable composition search one only needs to supply the atom types (i.e., the chemical elements) and USPEX can then, in principle, identify both the stable stoichiometries and the corresponding structures. The calculations were carried out over the course of 80–100 iterations (“generations”). Starting from an initial population of 120 structures, randomly produced using space group symmetry, which sample 20 different compositions in the first generation, each subsequent generation was populated by structures obtained by applying the so-called variation operators – heredity, mutation and permutation. The heredity operator produces a new structure by matching slices (chosen in random directions and with randomly shifted atomic positions) of two parent lattices. This operation greatly increases diversity and was set to provide 50% of the new members in a generation. A further 10% of newly generated lattices were produced randomly from the crystal space groups. The remaining 40% were obtained from mutation operations: 10% each from permutation (i.e., the random exchange of chemical types between a pair of atoms), transmutation (i.e., randomly chosen atoms are transformed into another chemical species present in the system), soft mutation (i.e., creating a new structure by large atomic displacements along the eigenvectors of the softest phonon modes), and lattice mutation (i.e., a new lattice is obtained by applying a distortion defined by a symmetric strain matrix on the old lattice). For several ambiguous alloy structures, a fixed composition search, mainly with a variable number of “molecular” units, over 40–60 generations was carried out with a population size of 20–30 structures per generation.

Trial structures generated by USPEX have been fully relaxed and their final energies evaluated based on first principles density functional theory (DFT). The density functional computations were carried out using the Vienna ab initio simulation package (VASP)^21^,^22^. Here an iterative scheme was used to solve self-consistently the Kohn-Sham equations based on a residual-minimization technique combined with convergence acceleration through direct inversion in the iterative subspace to extrapolate the charge density mixing. A plane-wave basis set is employed to expand the valence electronic wave functions, while interactions with nuclei and core electrons are accounted for through the use of pseudopotentials. In the current work the projector augmented-wave (PAW) method^23^,^24^ was specially used. In addition, calculations with ultrasoft pseudopotentials (USP)^25^ have also been carried out for the Al-Ta system to verify the findings. For electron-electron exchange and correlation interactions the functional of Perdew and Wang (PW91)^26^, a form of the generalized gradient approximation (GGA), has been used in the current work. The relaxation of atom positions was performed via the action of a conjugate gradient optimization procedure over four stages. The first stage of relaxation was performed with “low” precision settings and crude convergence criteria. At the second stage the precision was set to “normal” and finer convergence criteria were employed. Both the first and the second stages employed the default energy cutoff for the planewave expansion, with the cell volume being held fixed, while allowing the cell shape to vary as the ions relaxed. The reason for the fixed volume setting was to prevent large forces on ions that were initially placed very close to each other from causing the cell to expand dramatically. In the third (fourth) stage the planewave energy cutoff was raised to 300 (400) eV, the cell volume was allowed to vary, and tighter convergence criteria were used. Following the four relaxation stages, the total energy was evaluated from a single point energy calculation converged to within 5 meV using a planewave energy cutoff of 500 eV. The reciprocal space resolution for k-points generation was set to 0.16, 0.12, 0.08, 0.06 and 0.04 2*π*Å^−1^ for the five calculation stages, respectively.

Vibrational spectra were evaluated within the harmonic approximation using the Phonopy program^27^, using forces computed based on density functional theory as described above. Phonopy employs the Parlinski-Li-Kawazoe approach^28^ to generate supercell structures with finite displacements from the unit cell, fully considering the crystal symmetry. The force constant matrix of the system is determined by fitting the symmetry reduced elements of force constants to the linear relations between atomic forces and atomic displacements. A (*l* × *m* × *n*) supercell geometry (*l*, *m*, *n* = 2 or 3, depending on the unit cell size) has been employed with a 4 × 4 × 4 k-point grid, except for lattices with a hexagonal structure, where a 3 × 3 × 3 grid including the zone center was utilized.

## Results and Discussions

**Figure 1:**
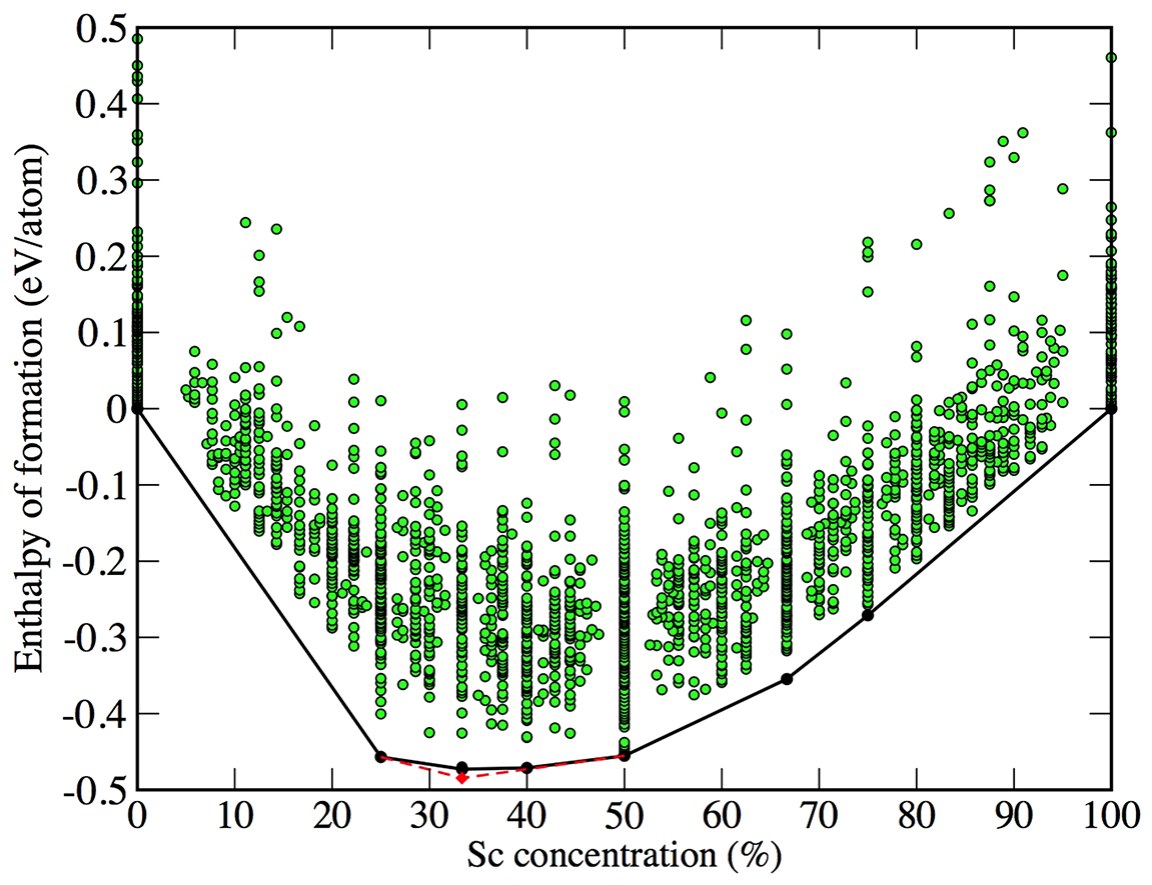
Illustration of the search for stable alloy phases in the Al-Sc binary system. The green circles indicate the formation enthalpy and composition of alloys found stable after each generation of the evolutionary algorithm search. The black line indicates the compositions and formation enthalpies of the final thermodynamically stable Al-Sc phases, forming the convex hull. The red dashed line shows the convex hull after the correct Al_2_Sc structure is added.

**Figure 2:**
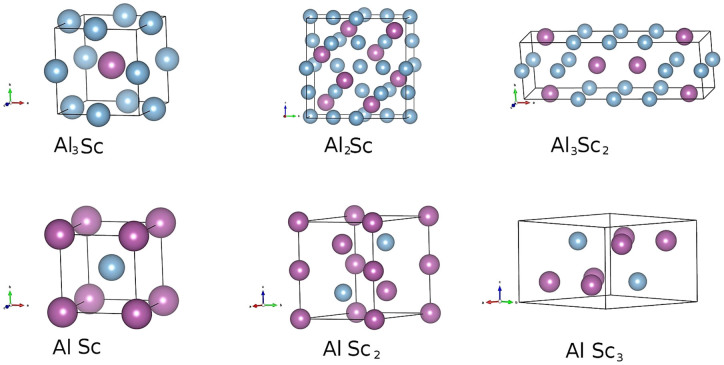
The unit cells and structures of the predicted stable Al-Sc phases given in Fig. **1.** The corresponding crystallographic information is given in Table 1. Al (Sc) are colored blue (purple).

**Figure 3:**
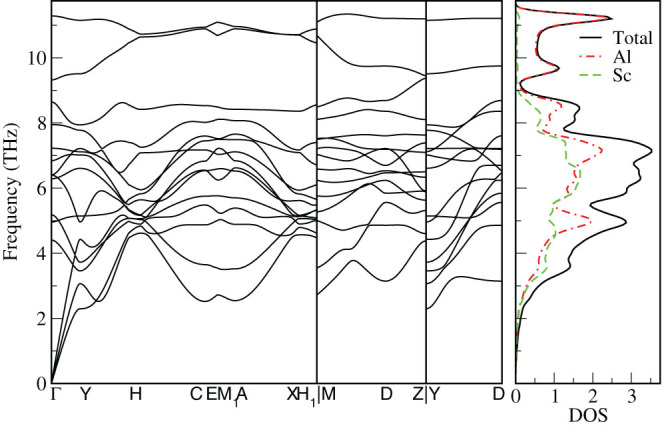
Left panel: the phonon dispersion lines along the three high symmetry directions of the Al_3_Sc_2_ Brillouin zone. Right panel: total and projected phonon densities of states.

**Figure 4:**
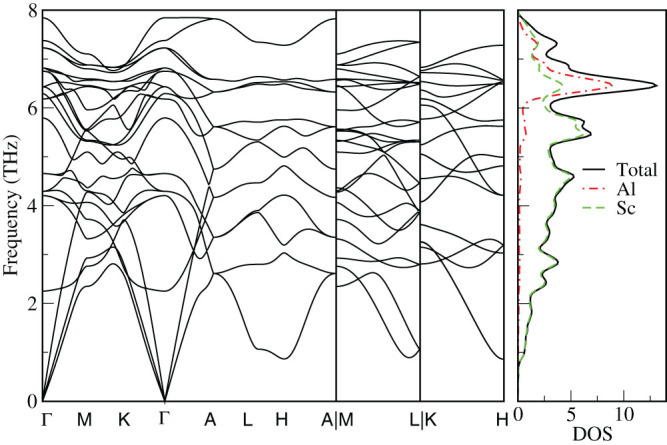
Left panel: the phonon dispersion lines along three high symmetry directions of the AlSc_3_ Brillouin zone. Right panel: total and projected phonon densities of states.

The enthalpies of formation for the Al-Sc phases identified as potentially stable after each generation of the USPEX structure prediction run are indicated in Fig. 1 by green circles. The enthalpy is given relative to the energy of the optimized elemental phases of Al and Sc, i.e., *E*(Al*_x_*Sc*_y_*) – [*xE* (Al) + *yE* (Sc)]/(*x* + *y*), where *E* stands for the total energy per atom of the phase, while *x* and *y* stand for the stoichiometric proportions of Al and Sc, respectively, in the binary phase. The compositions and formation enthalpies of the Al-Sc phases that are thermodynamically stable with respect to disproportionation to the adjacent phases at zero temperature and pressure form the so-called convex hull, indicated by the black line in Fig. 1 There are six points on the line, shown by the black circles, in addition to the two reference points for elemental Al and Sc. They represent the predicted stable binary phases: Al_3_Sc, Al_2_Sc, Al_3_Sc_2_, AlSc, AlSc_2_, and AlSc_3_. For the elements, Al and Sc, the correct fcc and hcp crystal lattices, respectively, have been found as the ground state structure. The optimized unit cells of the six Al-Sc phases predicted to be stable are shown in Fig. 2 and corresponding lattice parameters are given in Table 1. Three of the predicted alloys, i.e., Al_3_Sc, AlSc, AlSc_2_, exhibit crystal structures, shown in Fig. 2, that closely match those of the well known Al-Sc intermetallic phases with the same compositions^1^,^7^,^8^,^9^,^29^,^30^,^31^. In addition, there are several unexpected findings. Firstly, for the Al_2_Sc alloy an incorrect structure has been predicted, with a hexagonal crystal system instead of the expected C15 cubic lattice. Hence, to examine whether this discrepancy was a consequence of incomplete sampling of the configuration space, a fixed composition search was performed. To accelerate the resolution of this ambiguity, both the hexagonal and cubic structures were added to the initial population of randomly chosen structures. The C15 cubic structure, with a lattice parameter of 7.586 Å and space group number 227, shown in Fig. 2, was eventually found to be the most stable phase for the Al_2_Sc composition, having a lower formation enthalpy than the hexagonal phase by 15 meV/atom. The formation enthalpy of the correct structure is indicated by the red diamond in Fig. 1. Secondly, the intermetallic phase with the Al_3_Sc_2_ composition, while predicted to be thermodynamically stable by our calculations, has never been reported before. After the correct Al_2_Sc formation enthalpy is added to the plot, the Al_3_Sc_2_ point appears to be almost exactly on the straight line segment connecting the Al_2_Sc and AlSc points. Therefore any stability is likely to be marginal and the phase may not have been observed because it could easily disproportionate. Given the accuracy limits of the calculation methodology, it is worthwhile to further consider the prospects of a stable Al_3_Sc_2_ phase, in particular at elevated temperature, where thermal lattice vibrations and zero-point motion contributions to the free energy will have an effect on the relative phase stability. To investigate its crystal structure for possible phonon instabilities lattice dynamics calculations have been carried out. The primitive cell of the Al_3_Sc_2_ structure is a monoclinic crystal system (space group P2/m, number 10), half the size of the orthorhombic cell shown in Fig. 2. The phonon spectra along the high symmetry directions of the Brillouin zone (c.f. Fig. 16 in ref. 32) are given in Fig. 3, together with the total and projected density of states (DOS). Evidently, the phonon spectrum does not exhibit any instability in the form of “soft” modes with imaginary frequencies. Discussion of the influence of the vibrational contribution to the free energy will be given later. Finally, for the AlSc_3_ composition a stable phase has been identified with a hexagonal D0_19_ crystal structure, shown in Fig. 2. While experimentally this phase has not been reported, the prediction is in agreement with other recent computational investigations^33^,^34^. Furthermore, the phonon spectrum and the associated DOS, shown in Fig. 4, again suggest that the hexagonal crystal lattice is dynamically stable. The calculated elastic properties (elastic constants, bulk, shear and Young moduli, as well as Poisson ratios) are provided in Supplementary Table S1. Interestingly, several other computational studies^35^,^36^,^37^ have proposed a cubic L1_2_ structure for the AlSc_3_ phase. Given the hexagonal crystal systems of the adjacent stable phases AlSc_2_ and Sc, the stability of the cubic AlSc_3_ phase seems less likely. However, within the accuracy of current exchange-correlation functionals it is hard to be certain regarding the ordering of energies of closely related structures.

**Figure 5:**
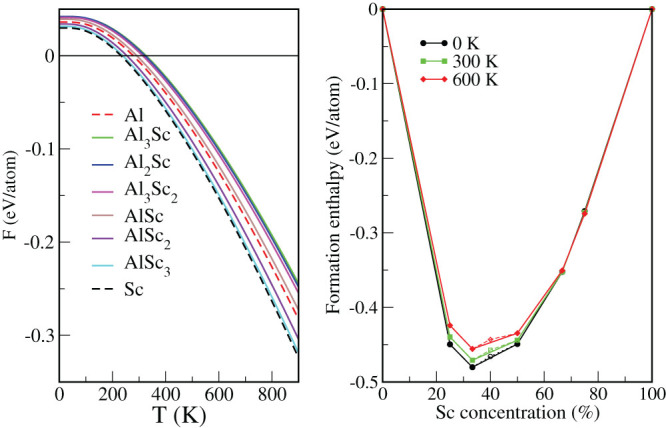
Left panel: the vibrational free energy for the potentially stable phases in the Al-Sc system. Right panel: the convex hull with zero point energy corrections and vibrational contributions at 300 and 600 K to the enthalpy. The dashed lines include the point for the Al_3_Sc_2_ phase.

Whilst in principle it is possible to conduct an evolutionary algorithm search with USPEX for finite temperatures and pressures to address the stability of alloy phases, computationally this would be a very costly and lengthy procedure. A more practical approach to investigating the phase diagram for mixing of two species is provided by the cluster expansion method^38^, provided the structure of the alloy is known. It can be effectively combined with semi-grand canonical Monte Carlo simulations^39^ to investigate the phase stability over a temperature and concentration range. However, even this approach becomes impractical for the whole Al-Sc diagram. Namely, the cluster expansion is an appropriate tool for the Al- and Sc-rich parts of the diagram, to address the stability of the isostructural solid solutions based around the fcc and hcp crystal systems, respectively. Given the different crystal systems of the Al_2_Sc, Al_3_Sc_2_ and AlSc phases with varying atomic coordinations in the middle of the diagram, a separate cluster expansion would be needed for each phase, which would not only be impractical, but would severely diminish the predictive power of the method. At low temperatures the dominant thermal contribution will be from the vibrational free energy, provided the enthalpy of ordering is appreciable, while the entropy of disorder will play an increasing role as the temperature rises. In the case of the Al_3_Sc (AlSc) compositions, for example, the eight (ten) structures that are related to the most favorable L1_2_ (B2) structure by permutations exhibit 0.17–0.24 (0.05–0.21) eV/atom higher enthalpies of formation than the L1_2_ (B2) phase. Therefore, at moderate temperatures one needs to consider only vibrational lattice excitations. The vibrational free energies, obtained from the phonon partition function, have been calculated to address the relative phase stability at elevated temperatures. The free energy *F* vs. temperature *T*, within the harmonic approximation, is shown for each Al-Sc phase in the left panel of Fig. 5. It is clear that the vibrational contributions do not help to stabilize the Al_3_Sc_2_ phase at elevated temperatures, since the slope of the AlSc free energy line is visibly more negative than that of either Al_2_Sc or Al_3_Sc_2_. The enthalpy of formation for the six Al-Sc phases, corrected for the zero point vibrations, is shown in the right panel of Fig. 5. The same plot also shows the room temperature (300 K) enthalpy of formation and at 600 K. At all temperatures considered the free energy of formation for Al_3_Sc_2_ (i.e., the points on the dashed lines) lies slightly above the convex hull (full lines). In fact, as the temperature rises, this point becomes further above the hull. Therefore, the conclusion is that the predicted Al_3_Sc_2_ phase is thermodynamically unstable with respect to disproportionation, albeit marginally so. In contrast, the stability of the predicted AlSc_3_ phase increases with temperature. In summary, the evolutionary algorithm implemented in USPEX has correctly identified five previously known thermodynamically stable phases in the Al-Sc phase diagram and additionally predicted a further as yet unknown phase that is close to being stable at zero temperature and pressure within the uncertainty of the method. This finding confirms that the algorithm in combination with the underlying GGA DFT energy surface is reliable for the present phase diagram and therefore is likely to be suited to the exploration of stable structures in similar, but much less well characterized binary alloy systems, such as Al-Ta.

**Figure 6:**
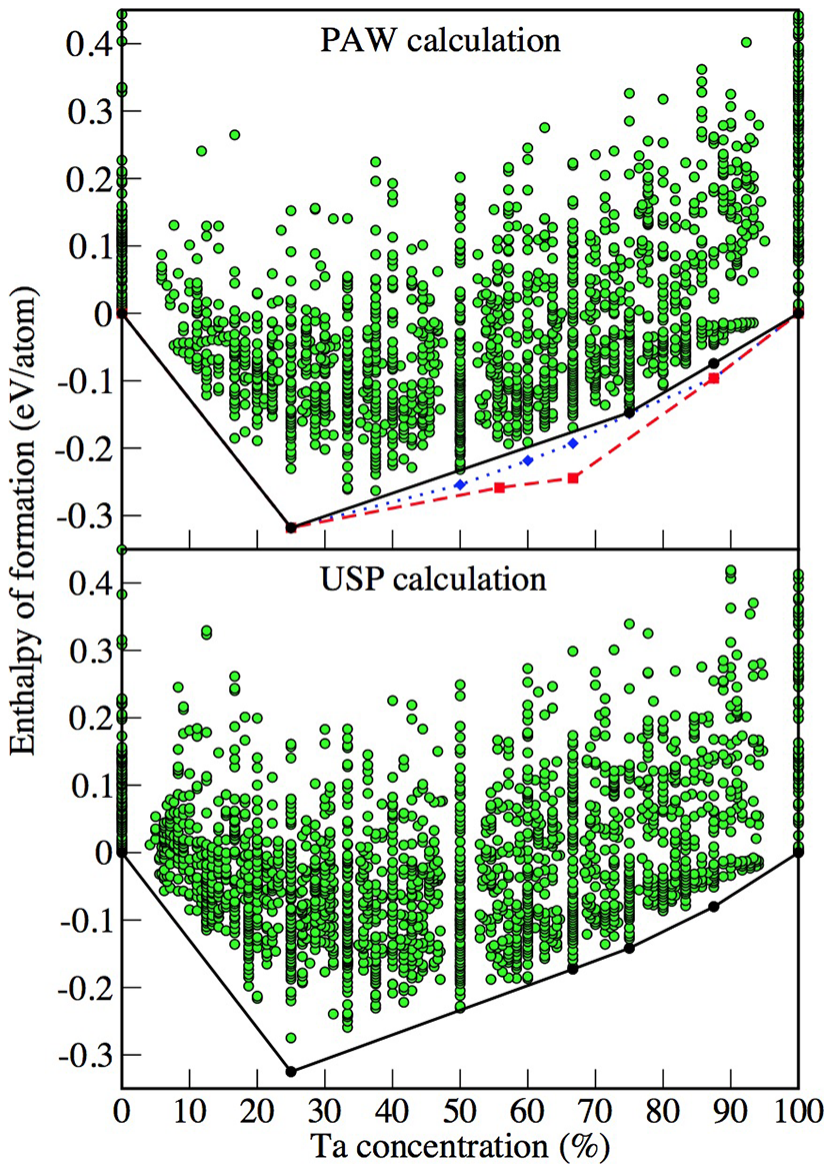
As in Fig. 1, for the Al-Ta system. Upper panel: First principles calculations using the PAW potentials. The dotted blue line and diamonds correspond to the results after performing fixed composition searches for the stable phases. The red dashed line and corresponding red squares indicates the results after explicit inclusion of previously known structures not found during the evolutionary search. Lower panel: As per the upper panel except using the USP potentials.

Based on the above, the same protocol as employed for Al-Sc has therefore been applied to the Al-Ta phase diagram. The formation enthalpies for the Al-Ta phases, identified as potentially stable after each generation, are shown in the upper panel of Fig. 6 by green circles. The compositions and formation enthalpies of the thermodynamically stable Al-Ta phases at zero temperature and pressure are indicated by the black convex hull line. There are three points on this line, shown by the black circles, in addition to the two reference points for elemental Al and Ta. The results given in Table 2 show that the crystal structure of the well known Al_3_Ta phase has been correctly reproduced. The structures of the other two intermetallics predicted here, with compositions of AlTa_3_ and AlTa_7_, have not been reported before. To verify the above findings, the same evolutionary algorithm search has been repeated, this time with the use of USPs (from the “hard” VASP library) instead of PAW to represent the combined potential of the core electrons and nuclei. This further run serves two purposes. Firstly, the evolutionary algorithm is an incomplete search through configuration space that involves random numbers. By performing a second run there is the possibility of sampling further configurations that may not have been identified in the first run. Secondly, the pseudopotential approximation and the choice of associated parameters can lead to some variation in the quality of the results. Hence, this further run aims to verify whether the findings are likely to be robust in regard to this factor. The results of the repeated search using the USPs are shown in the lower panel of Fig. 6. Despite the change in the pseudopotential and repeated evolutionary search the findings are in excellent agreement with those from the PAW calculations. The same binary phases, Al_3_Ta, AlTa_3_ and AlTa_7_, have been identified as stable, with practically identical structures and similar formation enthalpies. The only notable change is the finding of a stable phase with a composition of AlTa_2_ in the USP-evaluated diagram. However, both sets of results are not actually that different in this region of the phase diagram. It just happens that for the USP calculations this phase falls slightly below the tie line between the adjacent phases, whereas in the PAW case the corresponding energy is situated just above the same line. Whatever the precise quantitative position might be, it is unlikely that the AlTa_2_ phase will be particularly stable with respect to disproportionation. Nevertheless, one cannot completely discard the possibility of this and other potentially stable compositions, such as AlTa, which in both calculations almost falls on the convex hull. For this latter phase in particular, configurational entropy might play an important role since the number of possible states would reach a maximum for a disordered phase of this composition.

Another important, but less obvious difference between the two sets of results is the finding of a monoclinic crystal system as the stable Al phase in the USP calculations instead of the correct fcc lattice. This is not entirely unexpected, considering previously reported problems with some other similar pseudopotentials^40^,^41^. Based on this, and the underlying nature of the formalism, it can be presumed that the PAW results are the more accurate, at least for the present system.

In order to further confirm, as much as is possible, the relative stability of the structures of the Al-Ta intermetallics, fixed composition (with variable number of “molecular” Al*_x_*Ta*_y_* units) searches have been conducted for each concentration with a potentially stable structure. That is, based on the predictions from the variable composition search, structures with Al_3_Ta, AlTa, Al_2_Ta_3_, AlTa_2_, AlTa_3_ and AlTa_7_ compositions have been investigated separately. As the dotted line in the upper panel of Fig. 6 indicates, except for Al_3_Ta and AlTa_3_, new structures with lower formation enthalpies have been identified for all of the alloys. Despite the improvement over the variable composition search over the whole concentration range, there are still problems evident with the results. Firstly, an orthorhombic crystal structure (space group number 63), with twelve atoms in the unit cell, is predicted to be the stable form of the AlTa_2_ alloy. In contrast, at this composition a *σ*-phase (the FeCr prototype), with thirty atoms per tetragonal unit cell, features in virtually every phase diagram available in the literature^3^,^10^,^11^,^12^,^13^,^14^. In an effort to examine this apparent discrepancy, several fixed Al_10_Ta_20_ composition searches have been carried out, but all were unsuccessful at identifying the *σ*-phase. Even when all the structures in the initial random generation were restricted to the correct space group (number 136), an incorrect final structure was produced. However, when the actual crystal structure of the *σ*-phase, given in ref. 42, was added to the population of the initial randomly generated structures, the optimization and search confirmed it was indeed the most favorable phase at this concentration, with a formation enthalpy of −0.245 eV/atom. Secondly, for the equiatomic AlTa compound a tetragonal crystal structure (space group number 122), with sixteen atoms in the unit cell, is found to be the most stable. However, more recent experimental observations show that at the Ta-poor end the *σ*-phase is in equilibrium with a different, so-called *φ*-phase^15^,^16^,^17^. The *φ*-phase exhibits a complex Al_38_Ta_48_ stoichiometry^12^,^42^, accommodated by a monoclinic crystal lattice (space group number 14). Considering that the evolutionary algorithm repeatedly failed to predict the correct structure of the much simpler *σ*-phase, for the *φ*-phase no additional searches were performed. Instead, the initial structure, provided again in ref. 42. was simply evaluated over the same five steps of energy optimization, resulting in a favorable enthalpy of formation of −0.259 eV/atom.

**Figure 7:**
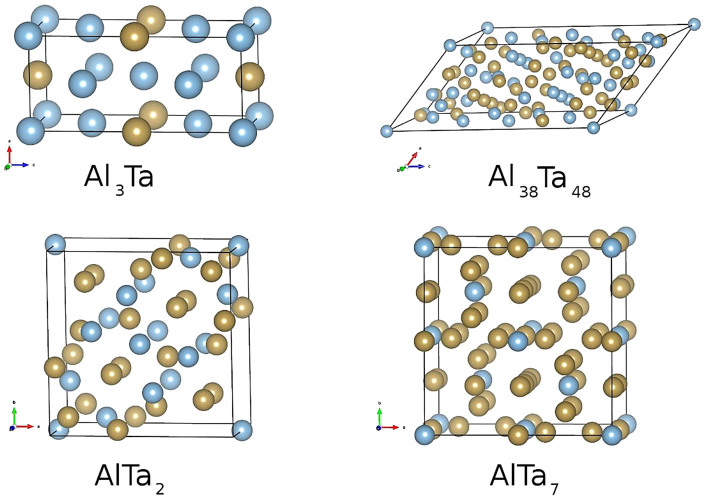
The unit cells and structures of the stable Al-Ta phases indicated in the upper panel of Fig. **6.** Here Al and Ta are colored blue and brown, respectively. The corresponding crystallographic information is given in Table 2.

The four Al-Ta phases with favorable formation enthalpies, i.e., Al_3_Ta, Al_38_Ta_48_, AlTa_2_ and AlTa_7_, are indicated by squares in the upper panel of Fig. 6. The dashed line connecting them suggests that, with the possible exception of Al_38_Ta_48_, they represent the thermodynamically stable phases. The figure is qualitatively (near quantitively, also) in agreement with Fig. 12 of ref. 12, which shows a similar plot obtained from CALPHAD modelling. This is not a surprise, since in our case the points for the *σ* and *φ* phases were evaluated starting from the experimentally observed structures, rather than the prediction from first principles. The structure of the optimized unit cell of each phase is shown in Fig. 7 and corresponding lattice parameters are given in Table 2. Their calculated phonon spectra (not shown) do not exhibit any “soft” modes with imaginary frequencies, indicating all the lattices are dynamically stable. It is interesting that the optimized crystal lattice parameters of the *φ*-phase deviate significantly from the experimentally determined values^16^,^42^ that provided the starting point for the optimization. In particular, the angle *β* relaxes substantially from the experimental value of ~ 100° to 49.4° (i.e., 130.6° with the alternative orientation of lattice vectors), associated with the elongation of the *c* lattice parameter to 19.344 Å (from 14.863 Å). The reason for this discrepancy is probably the fact that experimental samples, as reported^16^,^42^, exhibited near equiatomic, non-stoichiometric, composition and substitutional disorder. However, for the *σ*-phase the optimized cell parameters show little variation from the experimentally observed values, despite the reported large tolerance of this phase for non-stoichiometric composition and substitutional disorder^42^,^43^.

**Figure 8:**
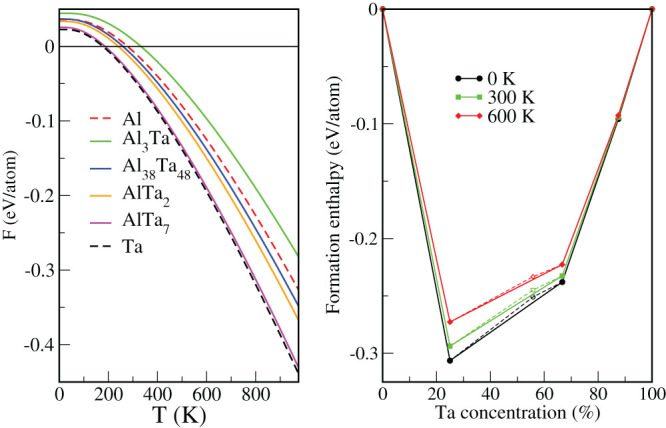
As in Fig. 5, for the Al-Ta system.

The *φ*-phase appears slightly above the convex hull segment between Al_3_Ta and AlTa_2_ in the upper panel of Fig. 6. This is similar to the segment between Al_2_Sc (with the corrected structure) and AlSc in Fig. 1. In the latter case, we have shown that, when the vibrational effects are taken into account, the predicted intermediate Al_3_Sc_2_ phase is rendered unstable. To address the relative thermal stability of the Al-Ta phases the vibrational free energies have been evaluated as functions of the temperature, shown in the left panel of Fig. 8. As for Al-Sc, the contribution of configurational disorder is neglected. The free energy slopes for the three Ta-rich phases are clearly more negative than that of Al_3_Ta. The corrected formation enthalpies of the Al-Ta phases with zero point vibration corrections, at room temperature and at 600 K are shown in the right panel of Fig. 8. Evidently, the convex hull segment between Al_3_Ta and AlTa_2_ remains below the Al_38_Ta_48_ point, suggesting that the *φ*-phase is unstable. Unlike that of the Al_3_Sc_2_ phase, however, its formation enthalpy shifts closer to the convex hull with rising temperature. At 1000 K (not shown) it is predicted to be stable, with the caveat that the harmonic approximation may not be appropriate any more and configurational entropy will undoubtly be important at such elevated temperatures. The crystal structures of Al_3_Ta, AlTa_2_, and the newly predicted AlTa_7_ intermetallic exhibit both the dynamic and thermal stability within the harmonic approximation.

The AlTa_7_ phase has not been reported before, probably due to the fact that it is at the less interesting, Al-poor, end of the diagram. The calculated elastic constants, moduli and Poisson ratio are provided in Supplementary Table S1. Considering that the Al contributes only 2.1% to the compound mass, making it technologically irrelevant, it is not a surprise that this seemingly stable phase has remained overlooked to date, similar to AlSc_3_. The failure to predict the structure and stability of the *σ* and *φ* phases directly from the evolutionary algorithm, while predicting that of AlTa_7_, is not surprising. Although AlTa_7_ in Fig. 7 appears to have a complex atomic structure, similar to AlTa_2_, the cubic cell with 64 atoms can be represented with a tetragonal unit cell with 32 atoms and a primitive cell with only sixteen atoms. In contrast, the crystal structure of the *σ*-phase of AlTa_2_ with 30 atoms per cell cannot be reduced to a smaller unit cell. It is more probable that any random search of configuration space will correctly sample smaller unit cells while it may miss larger and more complex arrangements. Hence, it appears from the present study that the structures of binary phases with up to ~ 20 atoms per primitive cell can be reliably predicted using the evolutionary algorithm within a reasonable computational effort. More complex structures and stoichiometries, such as Al_38_Ta_48_ appear to be beyond the current predictive power.

## Conclusions

Using the evolutionary crystal structure prediction algorithm, as implemented in USPEX, and the total energy from first principles calculations, stable intermetallic phases of Al with Sc or Ta have been investigated. In the Al-Sc case, the method has correctly identified four previously known thermodynamically stable phases: Al_3_Sc, Al_2_Sc, AlSc, and AlSc_2_. A hexagonal Sc-rich AlSc_3_ phase, previously only predicted, but not observed, has also been reproduced successfully. In addition, another phase with a Al_3_Sc_2_ composition has been found to be nearly stable at zero temperature and pressure. The relative structural and stoichiometric simplicity of the stable Al-Sc phases makes it possible for the algorithm to identify them in, practically, a single variable composition global search. In contrast, for the Al-Ta case repeated variable composition searches, followed by fixed composition searches, still failed to reproduce all the known stable phases and their structures. Only the Al_3_Ta phase, with a simple crystal structure, was correctly identified by these calculations, whereas the more complex structures of the *φ* and *σ* phases could not be found predictively within a reasonable effort. Hence, it is interesting that for the system that has been difficult to characterize experimentally, related problems also arise in the computational prediction. Nevertheless, a new cubic AlTa_7_ phase has been predicted to be stable at the Ta-rich end. The purely computational prediction of the existence of stable phases, AlTa_7_ and AlSc_3_, demonstrates the potential that the evolutionary algorithms may have in guiding materials research into alloys.

## Author Contributions

**Author contribution** The project was conceived by M.G., N.W. and K. McG, while the calculations were planned and performed by A.B. with contributions from J.G. The manuscript was initially drafted by A.B. and then added to and revised by all authors.

## Supplementary Material

Supplementary Information Supplementary Information

## Figures and Tables

**Table 1:** Crystallographic information for the predicted thermodynamically stable phases of Al-Sc and their formation enthalpies per atom

	space group		lattice parameters [Å]			
phase	name	number	a	b	c	enthalpy [eV/atom]
Al (fcc)	Fm 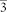 m	225	4.049			
Al_3_Sc (L1_2_)	Pm 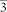 m	221	4.108			−0.457
Al_2_Sc^a^	P6/mmm	191	4.469		3.206	−0.473
Al_2_Sc (C15)^b^	Fd 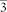 m	227	7.586			−0.485
Al_3_Sc_2_	Cmmm	65	13.882	4.367	3.110	−0.471
AlSc (B2)	Pm 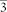 m	221	3.377			−0.455
AlSc_2_ (B8_2_)	P6_3_/mmc	194	4.891		6.165	−0.355
AlSc_3_ (D0_19_)	P6_3_/mmc	194	6.307		5.030	−0.271
Sc (hcp)	P6_3_/mmc	194	3.304		5.153	

^a^Incorrect structure predicted during the variable composition search.

^b^Corrected structure predicted by the fixed composition search.

**Table 2:** Crystallographic information for the predicted thermodynamically stable phases of Al-Ta and their formation enthalpies per atom

	space group		lattice parameters[Å]			
phase	name	number	a	b	c	enthalpy [eV/atom]
Al (fcc)	Fm 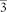 m	225	4.049			
Al_3_Ta (D0_22_)	I4/mmm	139	3.857		8.598	−0.318
Al_38_Ta_48_ (*φ*)	P2_1_/c	14	9.943	9.926	19.344	−0.259
AlTa_2_ (*σ*)	P4_2_/mnm	136	9.941		5.226	−0.245
AlTa_7_	F4_1_32	210	10.501			−0.096
Ta (bcc)	Im 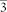 m	229	3.320			
