# Supplementary material for: Prediction of novel alloy phases of Al with Sc or Ta
Source: Sci Rep. 2015 May 7;5:9909. doi: 10.1038/srep09909 (PMC4423498; doi:10.1038/srep09909)
Supplement: Supplementary Information [file srep09909-s1.pdf]

Prediction of novel alloy phases of Al with Sc or Ta

A. Bilić, J.D. Gale, M.A. Gibson, N. Wilson and K. McGregor

Supplementary Information

TABLE S1. Crystallographic information for the predicted stable alloy phases $AlSc_3$ and $AlTa_7$, their elastic constants (C_{ij}), bulk (B), shear (G) and Young (E) moduli and Poisson ratios (ν).

alloy	$AlSc_3$	$AlTa_7$
space group	194	210
a [Å]	6.307	10.501
c [Å]	5.030	
C_{11} [GPa]	119	302
C_{12} [GPa]	45	121
C_{13} [GPa]	33	
C_{33} [GPa]	128	
C_{44} [GPa]	49	46
C_{66} [GPa]	37	
B [GPa]	65	182
G [GPa]		46
G_{yz} [GPa]	49	
G_{xy} [GPa]	37	
E [GPa]		232
E_x [GPa]	98	
E_z [GPa]	115	
ν		0.29
ν_{zx}	0.20	
ν_{xy}	0.33	
ν_{yz}	0.17	
